# Effects of N-acetylcysteine on the expressions of UCP1 and factors related to thyroid function in visceral adipose tissue of obese adults: a randomized, double-blind clinical trial

**DOI:** 10.1186/s12263-024-00744-7

**Published:** 2024-05-03

**Authors:** Mohammad Hassan Sohouli, Ghazaleh Eslamian, Seyed Hossein Ardehali, Seyed Ahmad Raeissadat, Ghazaleh Shimi, Katayoun Pourvali, Hamid Zand

**Affiliations:** 1 Department of Cellular and Molecular Nutrition, Faculty of Nutrition and Food Technology, Shahid Beheshti University of Medical Sciences, Tehran, Iran; 2 Department of Cellular and Molecular Nutrition, Faculty of Nutrition and Food Technology, National Nutrition and Food Technology Research Institute, Shahid Beheshti University of Medical Sciences, Tehran, Iran; 3 Department of Anesthesiology, School of Medicine, Shohada-e-Tajrish Hospital, Shahid Beheshti University of Medical Sciences, Tehran, Iran; 4 Physical Medicine and Rehabilitation Research Center Shahid Beheshti University of Medical Sciences, Tehran, Iran

**Keywords:** N-Acetylcysteine, Metabolism, thyroid hormones, obesity, UCP1, Adipose tissue

## Abstract

**Background:**

Evidences have shown that obesity is influenced by various factors, including various hormones such as thyroid hormones and the body’s metabolism rate. It seems that practical solutions such as weight loss diets and common drugs can affect these potential disorders. In this study, we investigate one of these common drugs, N-Acetylcysteine (NAC), on expressions of *UCP1* and factors related to thyroid function in adults with obesity.

**Methods and analysis:**

The current investigation was carried out as a randomized clinical trial (RCT) including 43 adults with obesity who were potential candidates for bariatric surgery. These individuals were randomly divided into two groups: 600 mg of NAC (*n* = 22) or placebo (*n* = 21) for a duration of 8 weeks. Visceral adipose tissue was utilized in the context of bariatric surgery to investigate the gene expression of *UCP1* and thyroid function. Polymerase chain reaction (PCR) was performed in duplicate for *UCP1, DIO2, DIO3, THRα* and *β*, and *18s* RNA (as an internal control) using the provided instructions to investigate the expression of the respective genes.

**Results:**

Our findings revealed that after 8 weeks compared to placebo, NAC caused a significant decrease in the expression of the *DIO3* gene as one of the genes related to thyroid function and metabolism. However, regarding other related genes, no statistically significant was found (despite the increase in *UCP1, DIO2*, and *THRα* expression and decrease in *THRβ* expression). In addition, after adjustment of possible confounders, no significant effect was observed on anthropometric factors and serum levels of thyroid hormones.

**Conclusion:**

The results of this study indicate that, following an 8-week period, NAC effectively decreases the expression of the *DIO3* gene in the visceral fat tissue, in comparison to the placebo.

**Supplementary Information:**

The online version contains supplementary material available at 10.1186/s12263-024-00744-7.

## Introduction

Obesity and overweight are recognized as a global epidemic and are spreading throughout the world [1]. Obesity, in particular, is recognized as a risk factor for acquiring non-communicable diseases [2, 3]. The causes of obesity have been attributed to environmental and genetic factors, as well as disruptions in energy balance, reduced basal metabolic rate, and changes in lifestyle [4]. On the other hand, numerous evidences have shown that obesity is related to hormonal disorders, for example, insulin resistance and hypothyroidism [5]. Obesity and thyroid dysfunction is a common disorder that frequently occurs among people, and a direct relationship between thyroid and obesity has been assumed [6]. Therefore, it seems that a practical solution including weight loss diets and supplements and common drugs can affect these potential disorders in people with obesity. Furthermore, there are several studies that suggest a link between adipose tissue thermogenesis and weight gain. There are two types of thermogenic fat cells, brown and beige adipocytes, that regulate energy balance [7]. It has been shown that the thermogenic protein UCP1 is associated with obesity-related metabolic diseases [8].

The search for pharmacological small molecules to induce browning of white adipose tissue is now a hot topic in obesity-related research. N-acetylcysteine (NAC) has recently garnered the interest of scientists. NAC is an amino acid that contains sulfur and is derived from acetylated cysteine [9]. The clinical use of this cysteine prodrug is due to its antioxidant property, free radical scavenger, and glutathione regeneration [9]. Moreover, there is compelling data indicating that NAC can effectively treat metabolic disorders, including obesity and metabolic syndrome (MetS) [9–11]. Various mechanisms have been suggested to account for these health advantages, such as the involvement of a group of enzymes called sirtuins (SIRTs), which are nicotinamide adenine dinucleotide (NAD+)-oxidized protein-dependent deacetylases and adenosine diphosphate (ADP) ribosyltransferases [12, 13]. SIRTs are overexpressed and activated during the “browning” or transdifferentiation of white adipose tissue (WAT) into brown adipose tissue (BAT) [14]. Uncoupling proteins (UCPs) are among the protein family whose expression increases in this transdifferentiation of adipose tissue [15]. UCPs, or uncoupling proteins, are transporters found in mitochondria that are involved in regulating body temperature and energy equilibrium. They are being investigated as a potential treatment for weight reduction. Three different isoforms of UCPs, namely UCP-1, UCP-2, and UCP-3, have been discovered in different tissues and the immune system [16, 17]. UCP expression levels are regulated by various factors, including dietary changes, free fatty acids (FFA), thyroid hormones, and transcription factors such as PPAR-γ and PPAR-α. These factors are involved in maintaining energy balance and regulating lipid metabolism [16–18]. UCP-1 in WAT decreases body fat, which is caused by an increase in energy expenditure in transgenic mice [19]. Nevertheless, there is a dispute over the involvement of UCP-2 and UCP-3 in thermogenesis. UCP-1 also has a role in fatty acid oxidation and influences glucose tolerance and insulin sensitivity [20]. Considering the potential role of NAC in ameliorating body adipose tissue, insulin resistance, and dyslipidemia, it is hypothesized that these beneficial effects of NAC may occur through increased UCP1 expression [21, 22].

On the other hand, NAC may have potentially beneficial effects on the level of hormones and the expression of thyroid-related genes by reducing oxidative stress and improving insulin resistance, which are clearly deranged in adults with obesity [23]. Some evidence suggests that thyroid hormone is regulated at the level of peripheral tissues through changing the expression of membrane transporters or intracellular deiodinases, which causes the conversion of pre-hormone T4 into active hormone T3 [24]. Iodothyronine Deiodinase type 1 and 2 (DIO1 and DIO2) are peripherally involved in this conversion. On the contrary, Iodothyronine Deiodinase type 3 (DIO3) converts T4 to reverse T3 and T3 to T2 through dioxidation of the inner ring [24]. On the other hand, evidence shows the role of these deiodinases and even receptors associated with thyroid hormones in obesity and insulin resistance [25]. So that the disruption in *DIO2* and thyroid hormone receptors (*THRα*) gene, unlike *DIO3* gene, caused a decrease in fat metabolism along with an increase in insulin resistance and visceral obesity, which indicates the beneficial function of this deiodinase to prevent the development of obesity [25, 26]. Hypoxia or ischemia caused by chronic inflammation such as obesity and metabolic syndrome can induce the re-expression of different types of deiodinase [25, 27]. This suggests that inactivating thyroid hormone through reducing the level of inflammation such as weight loss or antioxidant drugs such as NAC is important in these conditions (28).

Finally, induction of UCP1 and improvement of thyroid hormone function at the level of adipose tissue using NAC could probably be a suitable strategy along with common treatments for obesity, i.e. diet therapy and increasing physical activity in reducing obesity-related disorders. Therefore, the present study will be conducted with the aim of determining the effects of supplementation with N-acetylcysteine on the expression of *UCP1, DIO2 DIO3* genes as well as thyroid hormone alpha and beta receptors genes in visceral fat tissue of adults with obesity.

## Material & methods

### Participants

The present investigation is a double-blind randomized clinical trial (RCT) done from 2022 to 2023. It included persons with a BMI ≥ 35 kg/m^2^ (obesity) who were sent to Shohada & Modares Hospital in Tehran, Iran. The research sample was chosen from individuals who are candidates for bariatric surgery using convenience sampling, which was done based on specific criteria for inclusion and exclusion. In this study, fat samples were extracted during the abdominoplasty procedure to investigate the variables under study. The ethics committee of the Shahid Beheshti University of Medical Sciences approved the study (IR.SBMU.NNFTRI.REC.1402.012). Moreover, this clinical trial was registered on the Iranian Registry of Clinical Trials (www.irct.ir) website (IRCT20220727055563N2).

### Inclusion and non-inclusion criteria

The inclusion criteria encompassed the following factors: (1) The extent to which individuals are inclined to engage in and provide their consent by signing the informed consent document subsequent to attaining comprehensive understanding of the objectives and methods of the study; (2) This study includes individuals of both genders, aged between 25 and 50 years, who have a body mass index (BMI) equal to or greater than 35 kg/m^2^ and are classified as obese; (3) The participants in this research are adults who are being considered for bariatric surgery. Furthermore, individuals with the aforementioned conditions were excluded from participation in the study: (1) The historical background of various inflammatory, neoplastic, cardiovascular, hepatic, diabetic, renal, infectious, and gastrointestinal disorders; (2) Documentation of the utilization or historical utilization within the preceding three-month period of all categories of supplements or medications that exert an influence on hunger, body weight, or metabolic processes; (3) Receiving or following dietary and exercise treatments affecting weight during the last 6 months; (4) Alcohol intake and smoking.

### Exclusion criteria

In the event that the samples under investigation encounter any of the specified conditions over the course of the study, they will be disqualified from participation in the present research endeavor. (1) Any occurrence that has an impact on an individual’s state of health. (2) The act of consuming diverse supplements or medications that have an impact on body weight and metabolic processes, disregarding prior cautionary advice. (3) Noncompliance with medication adherence stemming from personal or external factors. The topic of immigration is being addressed. Furthermore, post-study, we assessed the acceptability and adherence of participants towards the intended medication. Individuals with an acceptance and adherence rate below 80% were eliminated from the study.

### Sample size calculation

The sample size for this study was determined by considering the difference in mean serum levels of T3 hormone, as indicated by previous research [23]. With a type I error probability level of 5% (α = 0.05) and a type II error probability level of 20% (β = 0.20, power 80%), the number of individuals required was calculated to be 20 subjects in each group. Given a potential loss rate of 10%, a total of 22 patients were included in each group.

### Study design and intervention

A total of 45 participants with obesity who satisfied the inclusion criteria for this double-blind, randomized clinical trial were randomly allocated to either the experimental group receiving NAC or the control group receiving a placebo. The intervention period lasted for a duration of eight weeks. Based on the findings of a meta-analysis conducted in this particular research domain, it was observed that the administration of NAC ranged from 600 to 1800 mg, while the intervention period varied from 5 days to 12 months. Notably, the study revealed that the majority of the drug’s efficacy was observed at doses below 1000 mg, with intervention durations typically lasting between 6 and 8 weeks. According to the literature, previous studies have indicated that there is no statistically significant variance observed when comparing doses ranging from 600 to 1000 across various factors. However, it has been observed that doses nearing 1000 can lead to a decline in patient adherence to the medication regimen. This decrease in compliance can be attributed to the increased frequency of daily drug administration. Ultimately, such non-adherence may potentially compromise the drug’s efficacy. Hence, a dosage of 600 mg was taken into account for the purpose of this investigation. In this investigation, a dose of 600 mg and an intervention length of 8 weeks were taken into consideration [9]. The duration of the intervention period in this trial spanned 8 weeks, during which both the intervention and control groups were administered the appropriate medications for a period of 6 weeks prior to undergoing bariatric surgery. The intervention group was administered a daily dosage of 600 mg of NAC, whereas the control group received a daily dosage of 600 mg of placebo (administered during lunch). The placebo was designed to resemble and taste similar to the NAC group, and was composed of starch powder. The administration of the tablets was facilitated by two pharmaceutical companies, namely Karen Pharmaceuticals, which provided placebo tablets, and Avicenna Pharmaceuticals, which supplied NAC tablets. At the onset of the study, participants were provided with pills and instructed to return the empty can packages at the conclusion of the six-week study period. This was done to assess the rate of drug acceptance. At the onset of the study, all participants were provided with recommendations to modify their daily total energy consumption. These recommendations were determined by calculating energy intake using age, gender, and BMI as determining factors. The calorie intake distribution was calculated to consist of 30% fat, with 7% being saturated fat and a maximum cholesterol consumption of 300 mg. Additionally, 50% of the caloric intake was attributed to carbohydrates, while the remaining 20% was allocated to protein. It is important to note that all participants were provided with identical dietary recommendations.

### Randomization and allocation

The assignment of BMI and sex variables was conducted using a stratified randomization approach, combined with the permuted block randomization methodology. This method involved the use of quadruple and binary blocks to guarantee an even distribution of these variables throughout the different groups. The quadruple block or double block designs were constructed using a web platform, with the sample size consisting of 45 patients (source: www.sealedenvelope.com).

The pharmaceutical boxes were assigned unique codes in order to ensure concealment during the randomization process. Additionally, the software is capable of generating the desired code. The allocation concealment strategy ensures that neither the participants nor the researchers are aware of which group received the NAC or the placebo. The company responsible for receiving the NAC and placebos incorporated the corresponding codes onto the packaging materials. Each participant in the trial was provided with a pharmaceutical package, which was allocated based on the predetermined sequence. The generated sequence utilized for the study was characterized by its inherent unpredictability.

### Anthropometric and physical activity measurements

Anthropometric parameters were assessed both before and after the trial. The height and weight of adults were assessed while wearing little clothing and without shoes. The weight of each participant was measured twice using the Seca digital scale, which is made in Germany and has a precision of 0.01 kg. The researchers employed the International Physical Activity Questionnaire (IPAQ) to assess the level of physical activity at both the beginning and conclusion of the study [28].

### Dietary assessment

To assess the nutritional intake of patients, including energy, macronutrients, micronutrients, and caffeine, a 24-hour dietary recall questionnaire was administered at the start and end of the study. This questionnaire covered one day off and two non-holiday days for each participant, resulting in a total of 9 food notes. The questionnaire was conducted through face-to-face and telephone interviews. The dietary records were analyzed using Nutritionist IV (N4) nutritional software.

### RNA extraction, cDNA synthesis, and quantitative real-time polymerase chain reaction

Adipose tissue extracted from visceral adipose were subjected to total RNA extraction using the RNX-Plus solution and following the directions provided by the manufacturer (Cinaclone, Iran). In addition, in accordance with the guidelines provided by the manufacturer, a quantity of 1 µg of total RNA (Viragen, Iran) was utilized for the synthesis of cDNA. The polymerase chain reaction (PCR) was conducted in duplicate for *UCP1, DIO2, DIO3, THRα* and *β*, and *18s* RNA (as an internal control) using the provided instructions. The final volume of the reaction was 20 µL. (1) 10 µL BIOFACT™ 2X Real-Time PCR Mix Master (for SYBR Green I; High. Rox, BIOFACT, South Korea); (2) 7 µl double-distilled water; (3) 0.5 µl each of forward and reverse primer (10 pmol/µL); (4) and 2 µl cDNA. 40 cycles of amplification were performed following a 15-minute at 95 °C initial denaturation stage. There was one denaturation step and one annealing step for each two-step cycle. The denaturation steps for *UCP1* and *DIO2* included 25 s at 95 °C and for the rest of the genes (*DIO3, THRα and β*) included 15 s at 95 °C. In addition, the annealing step for *UCP1* and *DIO2* included 25 s at 60 °C, for *DIO3* and *THRα* included 25 s at 52 °C, and for *THRβ* also included 25 s at 54 °C. The temperature range of the melt curve was between 60 and 95 °C, as measured using the StepOnePlus Real-Time PCR instrument from Applied Biosciences in Paisley, UK. The results for gene expression were obtained using the fold change, as indicated by the formula 2^−ΔΔct^. Table S1 contains a comprehensive list of the utilized primers.

### Thyroid hormones measurements

Thyroid hormones including thyroid-stimulating hormone (TSH), free triiodothyronine (FT3), and free thyroxine (FT4) were measured by ELISA (Pishtaz Teb, Tehran, Iran). For TSH test, Intra-assay and inter-assay coefficient of variation (CV) was 1.5 and 1.9%, respectively. FT4 intra- and inter-assay CV were 4.7 and 4.9%, respectively. FT3 intra- and inter-assay CV were 5.2 and 3.9%, respectively.

### Statistical analysis

The qualitative aspects were represented numerically using percentages, while the quantitative data were reported as the mean accompanied by the standard deviation. An independent sample T-test was employed to compare the means of the quantitative variables and the means of their changes between the two groups at the start and end of the study. A paired sample T-Test was utilized to compare the mean of quantitative variables before and after the intervention within each group. The qualitative characteristics of the two groups were compared utilizing either the chi-square test or Fisher’s exact test. Following the consideration of potential confounding variables, the impact of NAC on quantitative variables was assessed through the implementation of the covariance test, also known as analysis of covariance (ANCOVA). Statistical analyses were conducted using SPSS software version 16, with a significance level of *P*-value < 0.05 being deemed as statistically significant.

## Results

### Participant characteristics

**Fig. 1:**
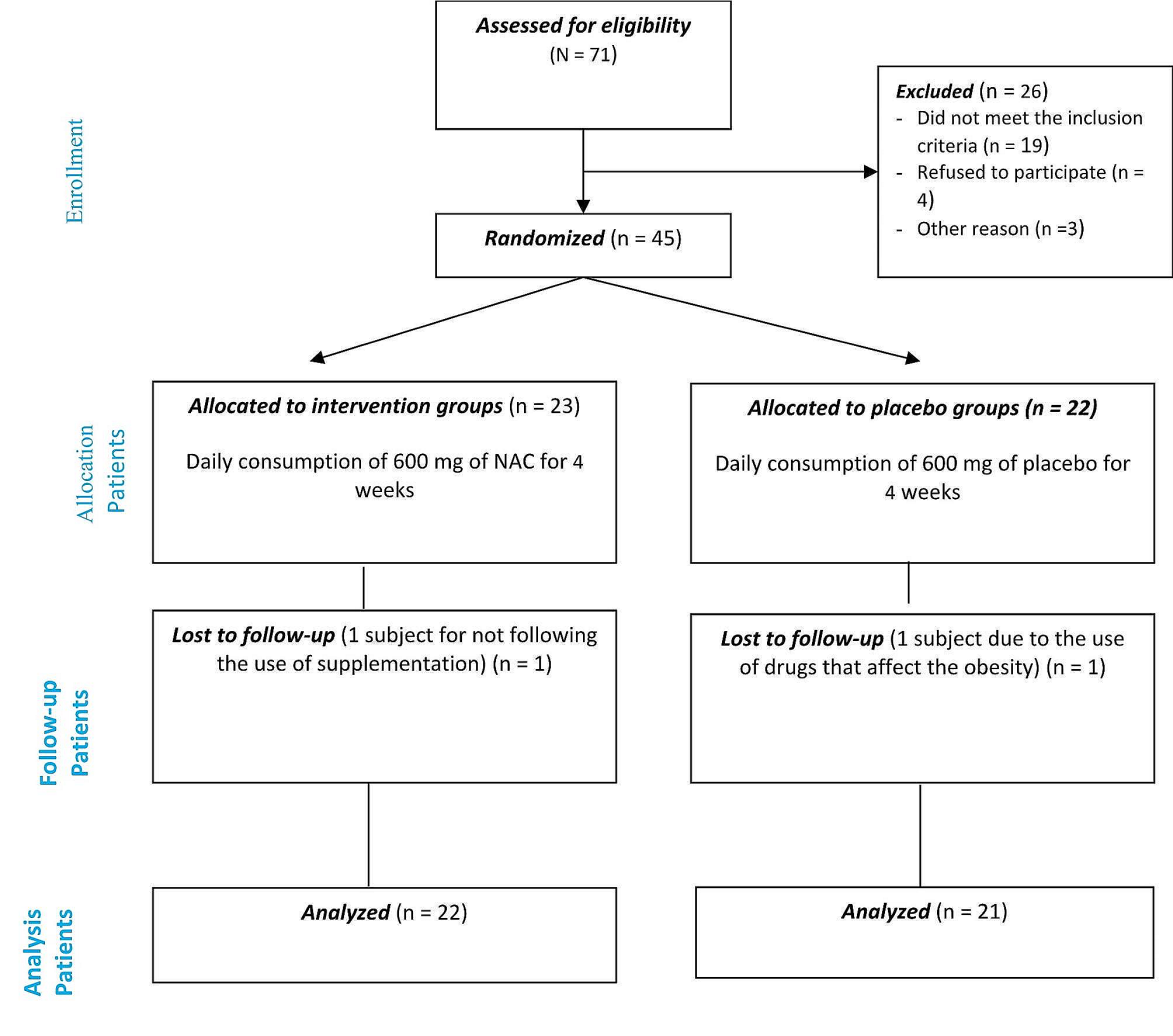
Consort flow diagram for the trial

Out of the 45 adults who were eligible for inclusion, 43 were chosen for the final analysis. Among them, 22 were in the intervention group and 21 were in the placebo group (Fig. 1).

Table 1 presents the starting demographic and metabolic factors of the subjects. The average ages of the participants in the placebo group were 40.18 years with a standard deviation of 6.32, while in the NAC group, the average age was 41.09 years with a standard deviation of 5.25. There was no statistically significant difference between the two groups in terms of characteristics such as gender, anthropometric parameters (weight, BMI, WC), thyroid hormones (FT3, FT4, and TSH), physical activity, multivitamin intake, drug use, marital status, and education level.

**Table 1:** Baseline characteristics of participants

Variables	Groups, mean (SD)		*P*-value^a^
	*NAC (n = 22)*	*Control (n = 21)*	
Age, y	41.09(5.25)	40.18(6.32)	0.598
Female (n, %)	12 (54.5)	11 (52.3)	0.609
Weight (kg)	125.02(12.12)	126.88(11.60)	0.810
BMI (kg/m^2^)	44.36(3.04)	44.03(4.09)	0.609
Waist-circumference (cm)	137.55(11.07)	136.27 (10.39)	0.936
FT3 (pmol/L)	4.47 (0.59)	4.51(0.57)	0.825
FT4 (pmol/L)	14.82(1.29)	14.87 (1.12)	0.894
TSH (mIU/L)	2.23(0.49)	2.35(0.66)	0.547
Physical Activity (met.h/wk)	1221.00(651.03)	1199.91(741.21)	0.981
Multivitamin use (n, %)	6(27.2)	6(28.5)	0.990
Drug use (n, %)	9 (40.9)	10 (47.6)	0.498

^a^ Data obtained from independent sample T-Test for continuous variables and Chi-square for categorical variables

Abbreviations: NAC: N-Acetylcysteine, BMI: Body mass index, FBS: Fasting blood sugar, HDL-C: High density lipoprotein-cholesterol, HOMA_IR: Homeostatic model assessment-insulin resistance, LDL-C: Low density lipoprotein-cholesterol; TC: Total cholesterol, TG: Triglycerides, WC: Waist circumference, hs-CRP: high-sensitivity C-reactive protein

#### Dietary intake

The dietary intake is specified in Table 2. Table 2 presents the dietary intake. Based on the findings of the 24-hour meal recall questionnaire and a comparison between the initial and final stages of the study, it was seen that there was a substantial increase in the consumption of vitamin C in both groups. Nevertheless, there was no notable disparity in the average intake of calories and other essential nutrients among the groups at both the beginning and conclusion of the experiment.

**Table 2:** Energy, macronutrient, and micronutrients intake at baseline and at the end of study

	NAC			Placebo			*P*-value^b^
	Baseline	After	*P*-value^a^	Baseline	After	*P*-value^a^	
Energy (kcal/d)	3705.29(187.18)	3679.43(164.14)	0.066	3710.67(183.67)	3691.59(156.46)	0.175	0.817
Carbohydrate (g/d)	544.55(78.88)	504.01(71.50)	0.125	555.38(76.09)	525.96(72.09)	0.177	0.233
Protein (g/d)	137.50(35.75)	137.96(29.36)	0.670	130.95(16.74)	129.18(17.63)	0.169	0.174
Fat (g/d)	128.72 (30.31)	126.72(31.96)	0.098	120.03(29.92)	118.96(27.76)	0.254	0.165
SFA (g/d)	40.50(11.65)	40.31 (8.84)	0.950	40.62(13.38)	45.74(9.40)	0.141	0.075
MUFA (g/d)	43.28(10.24)	41.23(13.39)	0.507	39.85(11.16)	47.72(21.89)	0.141	0.278
PUFA (g/d)	28.77(8.82)	27.31(7.33)	0.604	26.19(11.20)	24.89(10.81)	0.629	0.508
Fiber (g/d)	56.11(30.74)	56.63(14.21)	0.952	59.93(26.50)	47.94(19.09)	0.122	0.121
Sodium (mg/d)	5933.60(2496.40)	5780.27(1786.81)	0.790	6797.52(4676.22)	5164.25(2299.73)	0.229	0.363
Vitamin B12 (mcg/d)	5.71(2.94)	6.95(3.40)	0.149	6.79(2.57)	9.86(7.91)	0.137	0.150
Folate (mcg/d)	659.09(200.59)	614.37(113.68)	0.435	789.98(204.53)	699.07(159.87)	0.101	0.338
Magnesium (mg/d)	588.84(174.66)	553.24(107.96)	0.482	597.95(116.58)	532.87(122.81)	0.148	0.591
Calcium (mg/dl)	1596.34(418.94)	1389.14(407.04)	0.154	1733.79(305.62)	1673.61(459.80)	0.631	0.058
Vitamin A (RE)	1386.65(598.11)	1066.93(479.83)	0.315	1099.95(660.08)	1258.34(856.25)	0.479	0.401
Vitamin E (mg/d)	15.52(6.39)	19.22(9.14)	0.109	15.31(4.15)	18.71(5.95)	0.211	0.347
Vitamin C(mg/d)	266.74(164.61)	302.91(125.18)	**0.045**	260.43(143.31)	296.89(83.45)	**0.040**	0.848
Vitamin D (mcg/d)	2.34(1.45)	2.85(3.17)	0.481	2.91(2.15)	2.57(1.83)	0.268	0.471
Selenium (mg/d)	127.72(120.93)	153.83(57.47)	0.469	157.91(108.65)	128.59(48.92)	0.279	0.154
Zinc (mg/d)	18.58(4.99)	17.33(3.72)	0.446	18.35(2.59)	17.15(4.38)	0.276	0.896

^a^ Data obtained from independent sample T-Test.

Data are expressed as Mean (SD).

a: *P*-values for comparison of within-group differences

b: *P*-values for comparison of mean values between two groups

PUFA: Polyunsaturated fatty acid, SFA: Saturated fatty acid, MUFA: Monounsaturated fatty acid

#### Findings of UCP1, DIO2 and DIO3, THRα, and THRβ gene expressions

Upon doing an analysis of real-time PCR data and examining the fold change in gene expression, our results indicate that the level of *DIO3* gene expression in the intervention group, who consumed NAC for 8 weeks, was considerably lower than the control group who received a placebo (*P* < 0.001; fold change: 0.17). Our research results indicate that there was no significant impact on the expression of *UCP1* (P 0.404; fold change: 2.20), *DIO2* (P 0.412; fold change: 2.36), *THRα* (P 0.606; fold change: 1.80), and *THRβ* (P 0.899; fold change: 0.83) genes following the administration of NAC compared to a placebo. Although the expression of *UCP1, DIO2, THRα* genes except *THRβ* gene increased in the intervention group compared to the control group (Fig. 2a and 2b).

**Fig. 2a:**
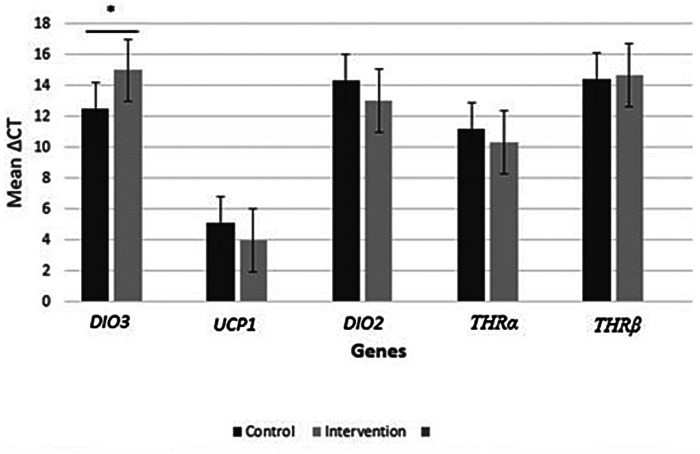
ΔCT mean values of *DIO3*, *UCP1*, *DIO2*, *THRα*, and *THRβ* gene expressions between control and intervention groups. There was a significant difference in ΔCT mean values of *DIO3* between control and intervention group’s samples. Gene expression results were expressed as the fold change defined by 2^−ΔΔct^. The higher ΔCT values indicate lower gene expression. *P < 0.05 between control and intervention groups

**Fig. 2b:**
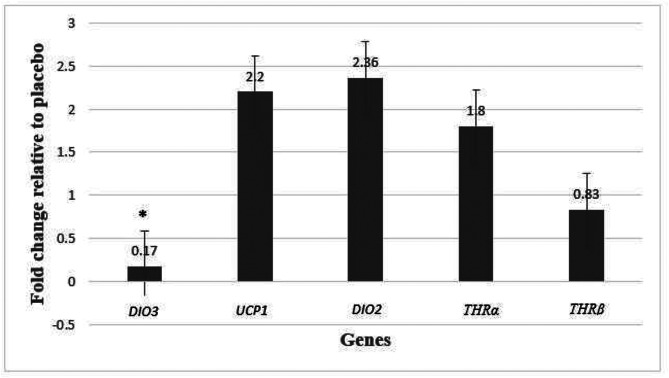
Gene expression results were expressed as the fold change defined by 2^−ΔΔct^. Also, ΔΔCT defined by ΔCT intervention group - ΔCT control group. *P < 0.05 between control and intervention groups

#### Thyroid hormones and anthropometry parameters

In both the control and intervention groups with NAC after 8 weeks, a significant decrease was observed in all evaluated parameters except the level of TSH hormones compared to the beginning of the study. However, compared to the placebo group (after adjusting for baseline values, energy intake, fiber, and vitamins E, A, D, C), no significant changes in FT3, FT4, and TSH concentration as well as anthropometry indices (weight, BMI, WC) were reported after NAC intake for 8 weeks (Table 3).

**Table 3:** Anthropometric characteristics and thyroid hormones at baseline and at the end of study

	NAC				Placebo				*P*-value^b^
	Baseline	After	Change	*P*-value^a^	Baseline	After	Change	*P*-value^a^	
Weight (kg)	125.02(12.12)	118.90(11.44)	-6.12 (2.01)	**< 0.001**	126.88(11.60)	121.39(11.51)	-5.49(2.68)	**< 0.001**	0.321
BMI (kg/m^2^)	44.36(3.04)	41.92(2.66)	-2.44 (0.41)	**< 0.001**	44.03(4.09)	41.82(3.78)	-2.21(0.69)	**< 0.001**	0.129
WC (cm)	137.55(11.07)	128.34(9.92)	-9.21 (2.99)	**< 0.001**	136.27 (10.39)	128.08 (9.88)	-8.19(1.92)	**< 0.001**	0.155
FT3 (pmol/L)	4.47 (0.59)	4.04(0.56)	-0.43(0.21)	**< 0.001**	4.51(0.57)	4.21(0.51)	-0.30(0.20)	**< 0.001**	0.059
FT4 (pmol/L)	14.82(1.29)	14.22(1.18)	-0.59(0.33)	**< 0.001**	14.87 (1.12)	14.43(1.08)	− 0.43(0.29)	**< 0.001**	0.065
TSH (mIU/L)	2.23(0.49)	2.20(0.43)	-0.03(0.12)	0.285	2.35(0.66)	2.22(0.39)	-0.13(0.54)	0.311	0.792

Data are expressed as Mean (SD).

^a^: *P*-values for comparison of within-group differences

^b^: *P*-value changes for between-group differences using analyses of covariance, considering **baseline values, energy intake, fiber, and vitamin E, A, D, C as covariate**

Abbreviations: NAC: N-Acetylcysteine, BMI: Body mass index, FT3: free triiodothyronine, FT4: free thyroxine, TSH: thyroid-stimulating hormone, WC: Waist circumference

## Discussion

The maintenance of energy balance as a preventive measure against obesity is contingent upon energy expenditure. Physical activity serves as the primary means of expending surplus energy. However, there exists a thermogenic system that has evolved to safeguard the body against hypothermia [29]. This system operates by uncoupling oxidative phosphorylation in brown adipocytes through the mitochondrial UCP-1 [29]. Previous studies have demonstrated that the augmentation of UCP-1 through genetic modifications or the administration of pharmacological substances can lead to a decrease in obesity and an enhancement in insulin sensitivity [29–31]. Furthermore, there is a strong association between obesity, particularly central obesity, and several endocrine disorders [32], such as thyroid dysfunction [33]. The lack of surprise stems from the fact that T3, as a regulator of energy metabolism and thermogenesis, holds a pivotal position in governing glucose and lipid metabolism, food intake, and the oxidation of fatty acids [33]. Thyroid dysfunction is correlated with alterations in body weight and composition, body temperature, as well as total and resting energy expenditure, regardless of physical activity levels [34]. Therefore, it seems that a practical solution including weight loss diets and supplements and common drugs can affect these potential disorders in people with obesity. Finally, according to the interpretations and evidence mentioned above, in this study, we investigated the effect of a potential anti-inflammatory and antioxidant drug called N-acetylcysteine on the expression of *UCP1* and also the expression of factors related to thyroid function.

The findings of the current RCT study showed that compared to the control group, NAC increases the expression of the *UCP1* gene by about 2.2 times, but the finding was not statistically significant. In addition, after adjusting for possible confounders, no significant difference was reported between the two intervention and control groups in terms of anthropometric parameters including weight, WC, and BMI. Although, to our knowledge, no study has been conducted to investigate the effect of NAC on *UCP1*, but considering the role of *UCP1* in increasing fat oxidation, increasing metabolism, and as a result, reducing weight, studies conducted to investigate the effect of NAC on anthropometric factors can also reveal some aspects of this relationship. In a meta-analysis consisting of seven RCT studies to investigate the effect of NAC administration on anthropometric factors (weight, BMI and waist circumference), the results of this analysis showed that NAC administration has no statistically significant effect on these variables [21]. Available evidence hypothesized that administration of NAC, a sulfur-containing antioxidant, may help weight loss in these individuals by regulating energy-related genes such as *UCP1*, as well as reducing insulin resistance and the anti-inflammatory pathway [21, 35]. In an animal study including mice fed with high fat diet (HFD) in order to investigate the effect of NAC on the expression of genes involved in energy expenditure, the results showed that the levels of activity and transcription of genes such as *UCP1, UCP3*, and *DIO2*, which are vital for cellular thermogenesis, increase significantly under the influence of NAC administration [10]. Also, in another animal study by Novelli et al., it was shown that the administration of this drug in mice fed with sucrose increases fat oxidation and energy expenditure of the whole body [22]. It seems that the effects of NAC on increasing fat oxidation and increasing *UCP1* activity are mediated by peroxisome proliferator-activated receptor gamma (PPAR-γ) transcription factors [10]. So, according to the evidence, one of the factors that can affect the level of *UCP1* expression is the transcription factors PPAR-γ and PPAR-α, which play a role in energy homeostasis and as regulators of lipid metabolism [17, 18]. Finally, In rodents, thermogenesis plays a much greater role in total energy expenditure, so the results cannot be extrapolated to humans [36]. Furthermore, the available evidence and studies about the effects of this drug on *UCP1* are limited to animal studies, and this issue can affect the proof and comparison of our findings. One of the important reasons for not observing statistically significant findings in our study, despite the high increase in the expression of *UCP1* and even other investigated genes, can be the relatively small sample size.

In addition, the findings of our study revealed that compared to placebo for 8 weeks, NAC caused a significant decrease in the expression of the *DIO3* gene as one of the genes related to thyroid function and metabolism. However, regarding other related genes, no statistically significant increase or decrease was found (despite the increase in *DIO2* and *THRα* expression and decrease in *THRβ* expression). In addition, no significant difference was reported between the two groups after adjusting for confounders regarding the concentration of thyroid hormones. Although receiving NAC compared to the control group caused a greater decrease in the concentration of FT3 and FT4, but these changes were not significant. Some of the evidence shows a correlation between higher obesity and increased T3 concentration, although other studies have not shown such a significant correlation [37]. For example, one study showed a decrease in T3 concentration in the visceral adipose tissue of people with obesity along with an increase in the activity of *DIO3* and a decrease in the activity of *DIO2* [25]. However, one of the causes of conflicting results in the level of thyroid hormones could be due to the measurement of total hormone concentration instead of free concentration. Conversely, empirical data supports the involvement of these deiodinases and even receptors associated with thyroid hormones in the development of obesity and insulin resistance. The disruption of the *DIO2* gene and thyroid hormone receptors (*THRα*) gene, in contrast to the *DIO3* gene, leads to a reduction in fat metabolism and an elevation in insulin resistance and visceral obesity [26, 38]. In line with this observation, it is worth mentioning that weight loss achieved by bariatric surgery increases the gene expression levels of *DIO2*, essential for adaptive thermogenesis, in beige adipose tissue of experimental models of diet-induced obesity [39]. These findings suggest that this particular deiodinase plays a crucial role in preventing the onset of obesity.

In a clinical trial study on 67 patients with acute myocardial infarction who were treated with NAC or placebo for 48 h, the results showed a significant decrease in reverse T3 levels after the intervention [23]. While no significant findings were observed on T3 level. Considering the role of DIO-3 in increasing reverse T3 and also the role of DIO-2 in converting T4 to T3, it seems that the findings of the mentioned study are in line with our results of a significant decrease in the expression of *DIO3*. It seems that the administration of NAC with its anti-inflammatory and antioxidant effects causes beneficial changes in the expression of genes related to thyroid function induced by hypoxia and inflammation [23]. Consistent with our results in a study on male Wistar rats with left anterior coronary artery occlusion, the results showed that NAC decreased the expression and activity of iodothyronine deiodinase type 3 in these rats [40]. These results showed that NAC prevents thyroid function disorders in these mice by restoring the redox balance [40]. Despite the incomplete understanding of the impact of NAC on the production and activity of *DIO3*, it is likely linked to the preservation of intracellular glutathione (GSH) levels and the restoration of redox homeostasis [41].

Evidence also suggests a role for the thyroid hormone receptor on insulin resistance and other metabolic disorders through reduced plasma levels of thyroid hormones in individuals with obesity [42]. In fact, the decrease in the plasma level of these hormones is associated with a decrease in the basic metabolism of the body and as a result, an increase in metabolic disorders [43]. Weight loss drugs have been shown to reduce the expression of these receptors in much the same way as adiponectin receptors or insulin sensitivity by reducing fat cells [42, 44, 45]. However, no study has been done to investigate the effect of NAC on this process and these receptors.

The primary advantage of this work lies in the innovative design of the randomized controlled trial (RCT), since it is the initial human investigation into the impact of NAC supplementation on metabolic genes in individuals with obesity. Furthermore, the inclusion of an investigation on visceral adipose tissue serves as an additional advantage of this study. Nevertheless, our findings are constrained by some limitations that provide different perspectives. Initially, our ability to evaluate obesity-related hormones and body fat percentage was limited by financial limitations. Furthermore, it is important to acknowledge that the correlation between RNA expression and protein expression may not always be consistent, which might be seen as an additional constraint in our study. But since genes such as *DIO3* are not normally expressed in adults, an increase in mRNA can indicate an increase in expression.

## Conclusion

The findings of the present study showed that after 8 weeks, NAC significantly reduces the expression of *DIO3* gene in the visceral fat tissue of individuals with obesity compared to placebo. However, it does not have a significant effect on expression of *UCP1*, *DIO2*, and thyroid hormone receptors as well as serum of thyroid hormone concentration. In general, due to the complexity of the effect of these genes on various metabolic processes and also due to the very limited studies conducted, various animal and human studies can provide a very important contribution in order to understand the beneficial effects of NAC.

### Electronic supplementary material

Below is the link to the electronic supplementary material.

Supplementary Material 1

## Data Availability

No datasets were generated or analysed during the current study.
