# Supplementary material for: Effects of N-acetylcysteine on the expressions of UCP1 and factors related to thyroid function in visceral adipose tissue of obese adults: a randomized, double-blind clinical trial
Source: Genes Nutr. 2024 May 3;19:8. doi: 10.1186/s12263-024-00744-7 (PMC11069202; doi:10.1186/s12263-024-00744-7)
Supplement: Supplementary file 1 — Supplementary Material 1 [file 12263_2024_744_MOESM1_ESM.docx]

***Table S1: List of human primer sequences***

| ***Human gene*** | ***Forward*** | ***Reverse*** |
| --- | --- | --- |
| UCP1 | GCCATCTCCACGGAATCAAA | CCTTTCCAAAGACCCGTCAAG |
| DIO2 | CAA ACA GGT GAA ATT GGG TGA | CAA GAA GGT GGC ATG TGG C |
| DIO3 | GAG GGT ATT GTA GTA AGG TGT ATT | AAA ACC CAA CCC ACC AAA TTC |
| THR-α | ATT CCT GCC CGA TTA CAT TG | ATG AAC TTG GTA AAC TTG CTG |
| THR-β | GTG TTA TTA GTT TGA TTA TTT GTT | CTA TTC CAC CAC TAT CCA C |
